# The Diagnostic Accuracy of Combined Enolase/Cr, CA125, and CA19-9 in the Detection of Endometriosis

**DOI:** 10.1155/2020/5208279

**Published:** 2020-09-02

**Authors:** Samaneh Rokhgireh, Abolfazl Mehdizadeh Kashi, Shahla Chaichian, Ali-Akbar Delbandi, Leila Allahqoli, Mahin Ahmadi-Pishkuhi, Sepideh Khodaverdi, Ibrahim Alkatout

**Affiliations:** ^1^Endometriosis Research Center, Iran University of Medical Sciences, Tehran, Iran; ^2^Pars Advanced and Minimally Invasive Medical Manners Research Center, Pars Hospital, Iran University of Medical Sciences, Tehran, Iran; ^3^Immunology Research Center, Immunology and Infectious Disease Institute, Iran University of Medical Sciences, Tehran, Iran; ^4^Department of Obstetrics and Gynecology, Kiel School of Gynaecological Endoscopy, University Hospital Schleswig Holstein, Campus Kiel, Arnold-Heller-Str. 3, Haus C, 24105 Kiel, Germany

## Abstract

**Background:**

The present study was designed to verify the accuracy of the noninvasive biomarkers enolase/Cr, CA125, and CA19-9 as a clinical diagnostic tool for the detection of endometriosis.

**Methods:**

A cross-sectional study was performed at Rasool-e-Akram Hospital affiliated to Iran University of Medical Sciences, Tehran, Iran, from April 2015 to April 2018. Eighty-six women were scheduled to undergo laparoscopy due to chronic pelvic pain, infertility, pelvic mass, and abnormal uterine bleeding. Serum and urine samples of all patients were collected preoperatively. Serum levels of CA125 and CA19-9, and urine levels of enolase-1 were measured. Serum levels of CA125 and CA19-9 were determined by the electrochemiluminescence method (ECL). Urinary enolase-1 was measured by the ELISA method.

**Result:**

Serum levels of CA125 and CA19-9 were significantly higher in the endometriosis group than in controls (*p* < 0.001, *p* = 0.004, respectively). Levels of enolase I and enolase/Cr were higher in patients with endometriosis, but the differences were not statistically significant. The sensitivity, specificity, positive predictive value (PPV), and negative predictive value (NPV) of combined enolase/Cr, CA125, and CA19-9 were 65%, 66.6%, 71%, and 60.1%, respectively. The positive likelihood ratio (PLR) and negative likelihood ratio (NLR) of combined enolase/Cr, CA125, and CA19-9 was 1.94 and 0.52, respectively. The area under the ROC curve for enolase/Cr + CA125 + CA19 − 9 was 0.675 (95% confidence interval 0.573-0.710).

**Conclusion:**

The present study revealed that concurrent measurement of enolase-1, CA125, and CA19-9 might be a valuable noninvasive test for the identification of endometriosis.

## 1. Introduction

Endometriosis is an estrogen-dependent disorder that severely affects the health and quality of life of 10% of women of reproductive age [1, 2]. Endometriosis causes infertility and debilitating symptoms such as dysmenorrhea, dyspareunia, dyschezia, and dysuria [3–5]. The gold standard for the diagnosis of endometriosis is direct visualization of a lesion in laparoscopy and histological confirmation of suspicious lesions in biopsy specimens [6]. The invasive nature of laparoscopy and the ambiguous symptoms tend to delay the diagnosis [7]. Due to the progressive nature of endometriosis, any delay in its diagnosis or treatment is liable to have severe consequences for the patient [8], including impairment of the patient's quality of life [9]. The greater burden of symptoms signifies much higher healthcare costs [10] and also reduces the patient's reproductive potential and fertility [11].

The discovery of a sufficiently sensitive and specific biomarker for nonsurgical detection of endometriosis would permit early diagnosis as well as prevent harmful sequelae of the disease [12]. As endometriosis is linked with a number of potentially confounding factors, the development of a noninvasive biomarker has been a challenging issue [13]. Despite various studies, no single biomarker or panel of biomarkers in peripheral blood or urine has been confirmed as a diagnostic test for endometriosis [14–16]. A review of the published literature suggests that an extremely high CA125 level combined with a high CA19-9 level is a possible indicator of endometriosis [17]. These tests may well be useful for the diagnosis of endometriosis, particularly deep infiltrating endometriosis (DIE) [18]. However, the power of the tests is considered low because they are unspecific. Besides, the tests are used to detect ovarian tumors as well [17, 19, 20]. In view of the fact that surgery is expensive and ridden with risks, and the validity of blood biomarkers has not been confirmed yet, some authors have investigated biomarkers in other body fluids such as saliva, peritoneal fluid [21, 22], and urine for their ability to detect endometriosis noninvasively [16].

A reliable urine examination could lead to the diagnosis of endometriosis without surgery or could eliminate the need for diagnostic surgery [16]. Elevated urinary enolase I levels have been claimed to be a determinant of endometriosis [12, 23], but other studies have shown that there is too little evidence to recommend any urinary biomarker alone for use in clinical practice [12, 16]. Some authors have employed a combination of biomarkers for the diagnosis of endometriosis [23]. Yun et al. reported that elevated urinary enolase, in conjunction with serum CA-125, may be used as a potential biomarker for endometriosis [23]. In view of the poor diagnostic accuracy of some biomarkers in the diagnosis of endometriosis [16], the present study was designed to verify the accuracy of the noninvasive biomarkers enolase/Cr, CA125, and CA19-9 as a clinical diagnostic tool for endometriosis.

## 2. Materials and Methods

This cross-sectional study was performed in Rasool-e-Akram Hospital, a tertiary care center, from April 2015 to April 2018. The investigation was approved by the committee of science and research ethics at the Iran University of Medical Science (IR.IUMS.REC 1395.95-01-204-27483).

Women who were candidates for diagnostic or operative laparoscopy due to an ovarian cyst, pelvic pain, infertility, suspicious endometriosis, or abnormal uterine bleeding were eligible for the study. The following inclusion criteria were used: (1) age 15-45 years; (2) no use of hormones or gonadotropin-releasing hormone agonists (GnRHa) during the preceding 3 months; (3) no underlying diseases such as infection, autoimmune disease, or cardiovascular conditions. Women who were diagnosed with gynecological diseases other than endometriosis (adenomyosis, malignancy, uterine polyp, acute or chronic inflammatory condition, endometrial hyperplasia) during surgery were excluded from the study.

General demographic data including age, weight, height, body mass index (BMI, kg/m^2^), marital status, gravidity, parity, abortion, smoking status, menstrual phase, menstrual pattern, and infertility history were either obtained directly from the patients or extracted from their medical records. The menstrual phase (proliferative and luteal phase) was determined on the basis of the published literature [24]. Urine and blood samples were taken from all patients preoperatively. For measurement of enolase-1, a urine sample (20 mL) was taken in a sterile plastic container after the induction of anesthesia and bladder catheterization. The urine sample was centrifuged at 1000 × g for 10 minutes in order to obtain sediment-free urine samples. The latter were stored at −70° Celsius until analysis [12]. The concentration of enolase-1 in urine was measured in ng/cc, using an ELISA kit according to the manufacturer's protocols (USCN Life Science & Technology Company, Missouri, TX), with a minimum detectable concentration of 0.312 ng/cc. Urine creatinine (Cr) levels were measured with commercial ELISA assays, and enolase-Cr levels were determined by the following formula: enolase-1/urinary Cr.

For measurement of CA19-9 and CA125 levels, blood samples were taken and centrifuged for 5 minutes with the serum withdrawn, and frozen at −80°C until analysis. Serum levels of CA19-9 and CA125 (U/mL) were measured using the electrochemiluminescence immunoassay on the Roche Cobas 6000 analyzer (Roche Diagnostics, Mannheim, Germany). All experiments were performed in a single laboratory.

**Figure 1:**
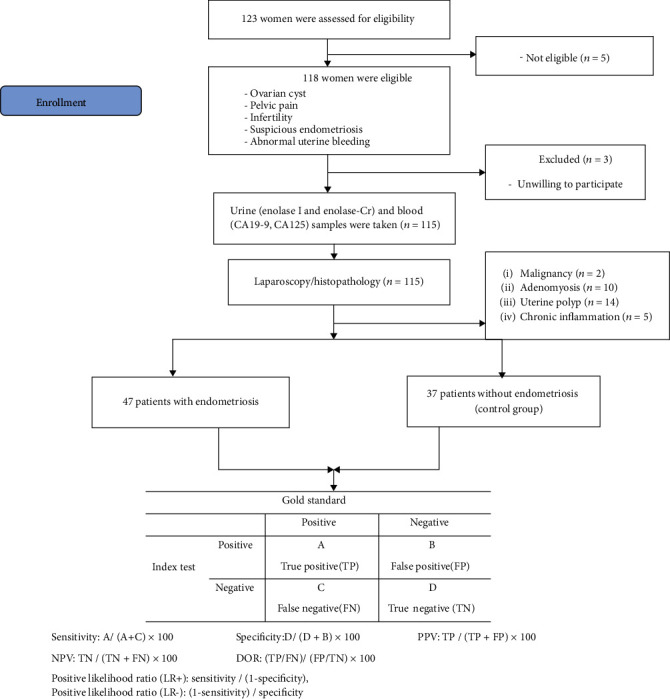
Diagram of the study.

All patients underwent laparoscopic surgery. Suspicious endometriotic lesions were excised during the operation and the patients were assigned to the endometriosis group or the control group after histological confirmation (Figure 1). The stage of endometriosis was determined according to the revised classification of the American Society of Reproductive of Medicine [25].

The primary outcome of the study was the diagnostic accuracy of the individual tests enolase/Cr, CA125, and CA19-9, and their combined application, as a clinical diagnostic tool for the determination of endometriosis. Associations between the patients' clinical characteristics and their biomarker levels were regarded as secondary outcomes.

### 2.1. Statistical Analysis

Commercially available software (IBM SPSS Statistics version 22; IBM, Chicago, IL, USA) was used for data analysis. Descriptive statistics (mean ± standard deviation) were used to present the data. The Shapiro-Wilk test showed that the data were not normally distributed. Data were also tested for geographical normality using the Q-Q plot and were found to be not normally distributed because of their nonlinear pattern. Therefore, nonparametric tests were used for analysis. The Mann-Whitney *U* test and the AVONA test were used to assess differences between the study groups. The chi-square test or the Fisher exact test was used to assess categorical variables. Diagnostic test analysis was applied to determine the sensitivity, specificity, positive predictive value (PPV), negative predictive value (NPV), positive likelihood ratio (PLR), and negative likelihood ratio (NLR) of the test. The usefulness of the test was established by the area under the ROC curve (AUC). Receiver-operator characteristics (ROC) of the combined marker (urinary enolase/Cr, and serum CA125, and CA19-9) were computed by plotting sensitivity vs. 1-specificity. All comparisons were based on a significance level of *p* < 0.05.

## 3. Results

Of 115 patients who underwent laparoscopy, 31 had a pathology of malignancy, adenomyosis, a uterine polyp, or chronic inflammation and were therefore excluded from the study. Endometriosis was confirmed in 47 patients and not confirmed in 37 patients. Women without endometriosis were regarded as controls. Ultimately, the data of 84 patients were eligible for analysis (Figure 1). No significant differences were observed between groups in regard to age, BMI, marital status, gravities, parity, abortion, smoking status, menstrual phase, menstrual pattern, infertility history, and urine creatinine levels. 61.7% (*n* = 29) of patients with endometriosis were operated in the follicular phase. Of 37 patients in the control group, 7 had a functional cyst, 6 had a dermoid cyst, 4 a hydrosalpinx, 7 a para ovarian cyst, 10 were infertile, and 3 patients had a serous cystadenoma. The patients' clinical characteristics are summarized in Table 1.

Urinary enolase-Cr was higher in patients with endometriosis than in controls, but the difference was not statistically significant (*p* = 0.106). Mean serum levels of CA125 were significantly higher in the endometriosis group than in controls (62.0 IU/mL versus 11.0 IU/mL) (*p* < 0.001). Likewise, mean serum levels of CA19-9 were higher in the endometriosis group than in controls (10.4 IU/ml versus 3.0 IU/mL). This difference was statistically significant (*p* = 0.004).

Urine and blood markers in patients with endometriosis and controls are shown in Table 2.

Sensitivity, specificity, PPV, NPV, PLR, and NLR were determined for the individual and combined biomarkers of endometriosis; the results are summarized in Table 3. The sensitivity of combined enolase/Cr + CA125 + CA19 − 9 (65%) was higher than that of enolase/Cr alone (44.3%). However, the NPV of enolase/Cr alone was higher than that of combined enolase/Cr + CA125 + CA19 − 9 (83.5% vs. 60.1%). The PLR and NLR of combined enolase/Cr + CA125 + CA19 − 9 were 1.94 and 0.52, respectively (Table 3).

An ROC analysis was performed to distinguish endometriosis from other conditions. The ROC analysis was also used to evaluate the diagnostic performance of combined enolase/Cr + CA125 + CA19 − 9.

**Figure 2:**
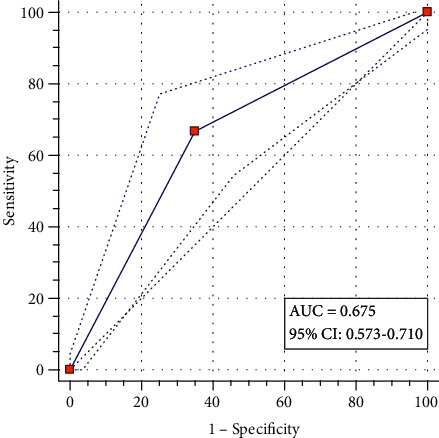
ROC-AUC curve for combined enolase/Cr + CA125 + CA19 − 9 as a test for the detection of endometriosis.

Table 3 shows the areas under the ROC (ROC-AUC) curves for the individual markers and combinations of biomarkers for all stages of endometriosis. Of the individual markers, enolase/Cr had an ROC-AUC of 0.443, and the combination of enolase/Cr + CA125 + CA19 − 9 had ROC-AUCs of 0.675 for all stages of endometriosis (Table 3 and Figure 2).

The ROC analysis was also used to determine the severity of endometriosis and evaluate the diagnostic performance of combined enolase/Cr + CA125 + CA19 − 9.

The ROC-AUC (0.763) for combined enolase/Cr + CA125 + CA19 − 9 showed that it was possible to distinguish between severe (stage III and IV) and mild (stage I and II) endometriosis (Table 4).

Associations between the clinical characteristics of patients with endometriosis and marker levels are shown in Table 5. Women with endometriosis had higher enolase/Cr levels in the luteal phase than in the follicular phase (21.8 ± 5.8 ng/mg Cr versus 11.3 ± 2.5 ng/mg Cr) (*p* = 0.039). The mean difference was -10.5 (95% CI -21.7-0.6).

There was no association between urinary enolase-Cr, CA 125, and CA19-9 levels on the one hand, and the patients' age, BMI, endometriosis stage, and infertility on the other hand (Table 5).

## 4. Discussion

Endometriosis has debilitating effects, including infertility and pain [3], which disrupts the patients' quality of life [23]. A sufficiently sensitive and specific biomarker for nonsurgical detection of endometriosis will enable clinicians to diagnose the condition early. The present study was designed to verify the accuracy of combined enolase/Cr + CA125 + CA19 − 9 as a diagnostic tool for endometriosis. A number of authors have reported on non-invasive biomarkers for endometriosis, determined from blood, urine, the eutopic endometrium, the peritoneum, as well as epigenetic markers of the endometrium [13, 14, 21, 23, 26–29]. The diagnostic accuracy of the tests has been shown in some studies but has not been conclusively proven yet [14, 21, 29].

The present investigation revealed higher levels of urinary enolase-Cr, CA-125, and CA19-9 levels in patients with endometriosis than in controls. In line with our findings, some studies have reported elevated serum levels of CA125, CA19-9 [17, 30, 31], and enolase-1 in women with endometriosis [12, 16, 23]. In Yun and coworkers' study, enolase-Cr levels were significantly higher in patients with endometriosis (1.25 ng/mg Cr vs. 0.75 ng/mg Cr) than in those without endometriosis. The underlying reason for increased levels of urinary enolase-1 is obscure [23]. Some authors state that the inflammatory process and hypoxia occurring in endometriotic lesions lead to hyper-expression of enolase-1 in renal tubules, causing secretion to urine [23, 27]. As for blood biomarkers, some studies have shown that inflammatory reactions in endometriosis lead to an alternation of endothelial permeability, and biomarkers enter the bloodstream. Thus, there will be a higher concentration of biomarkers in the ectopic endometrium than in the entopic endometrium [32].

Although in some studies have been reported that endometriosis is significantly associated with elevated serum CA19-9 and CA125 concentrations [15, 17, 33, 34], the diagnostic value of serum CA125 and CA19-9 concentration in endometriosis therefore remains unclear [30]. It has been reported that CA19-9 is elevated further in the more advanced stages of endometriosis [15]. The CA 19-9 is a pentasaccharide with carbohydrate ingredients including fructose components, and it belongs to a group of oncofetal antigens. In the fetal period, it is synthesized in the epithelial structures of the stomach, and its production is significantly decreased by adulthood. As well as recent studies report that CA 19-9 may be produced by glandular structures of the pancreas, gall bladder, bronchus, and some gynecological tumors [15]. Some studies show that CA-19-9 may be demonstrably elevated in endometriosis and show the same or decreased sensitivity as CA-125 [15, 17, 33].

In the present study, the sensitivity of enolase/Cr for identifying women with endometriosis was 97.8%, which was higher than the sensitivity of CA125 (69.5%), and much higher than the sensitivity of CA19-9 (27.5%). In Yun and coworkers' study, the sensitivity of normalized urine enolase-1 (enolase/Cr) was 76.9% for the diagnosis of endometriosis [23].

A wide range of data have been published about the sensitivity of enolase/Cr, CA125, and CA19-9 for the detection of endometriosis [18, 23, 30]. In the present study, the sensitivity of CA125 in the detection of endometriosis was 69.5%. CA 125 was reported to possess a higher sensitivity of 63% (95% CI, 42% to 77%) in the presence of severe disease. In the present study, the majority of women with endometriosis had stage III or IV disease. In line with previously reported data, we found that CA19-9 had the lowest sensitivity for the detection of endometriosis [24, 35].

Although urinary enolase 1, enolase/Cr, CA 125, and CA19-9 levels are increased in women with endometriosis, these parameters alone are reported to possess no diagnostic power [12, 14, 15, 23]. Therefore, in the present study, we used a combination of enolase-Cr, CA-125, and CA19-9 for the diagnosis of endometriosis. The sensitivity and specificity of the combined biomarkers in the detection of endometriosis were 65% and 66.6%, respectively. In line with our data, Yun and coworkers reported a sensitivity and specificity of 76.9% and 85.0%, respectively, for combined enolase I and CA-125 [23]. In the present study, the PPV and NPV of combined enolase-Cr, CA-125, and CA19-9 in women with endometriosis were 71% and 60.1%, respectively. The majority of researchers believe that the main parameters of a screening test are PPV and NPV. However, it should be noted that PPV and NPV depend on the population being tested and the technical characteristics of the screening test [25].

In the present study, although the diagnostic power of urinary enolase-Cr was lower than that of serum CA-125 for the detection of endometriosis, when combined with serum CA-125 and CA19-9, the AUC was increased to 0.675 (95% CI 0.573-0.710). Consistent with these data, in Yun and coworkers' study, the AUC of urinary enolase-Cr for the detection of endometriosis was 0.621 (95% CI 0.476–0.765), while the AUC of combined enolase I and CA-125 increased to 0.821 (95% CI 0.713–0.928) [23]. Any value between 0 and 1 is reported to be a good indicator of the accuracy of a test. The AUC value of 0.6-0.7 proved the sufficient diagnostic accuracy of the test [36]. Based on our data, we conclude that the combination of enolase/Cr, CA125, and CA19-9 is a sufficiently accurate and valid test for the detection of endometriosis.

The present study revealed that combined biomarkers possess greater sensitivity and specificity, and greater diagnostic power for the detection of endometriosis [23, 37, 38]. Urinary enolase-Cr may serve as one of several combined biomarkers for the detection of endometriosis in the future [16, 23].

As a secondary outcome, we noted that patients with endometriosis had significantly higher enolase-1 and enolase/Cr levels in the luteal phase than in the follicular phase. Analogously, Yun and coworkers reported significantly higher urinary enolase-1 and enolase/Cr levels in the secretory phase than in the follicular phase [23]. Further larger studies are needed to confirm the role of NNE in detecting endometriosis [16].

An ideal noninvasive test should have a high sensitivity and good specificity in patients with endometriosis. Easy and noninvasive detection of endometriosis would be a noteworthy achievement in preventing the progression of endometriosis, provide more options for planning the patient's treatment (medical or surgical), and help to determine the best time for administering the treatment. Although the present study yielded important data, it was limited by the small sample size. The data will have to be confirmed and proven in larger controlled studies.

## 5. Conclusion

The diagnosis and treatment of endometriosis are demanding because the symptoms vary. To date, laparoscopy is the gold standard for the diagnosis of endometriosis. Given the current ambiguity about various aspects of the condition, a period of 12 years may elapse between the onset of symptoms and the definitive diagnosis of endometriosis. It would be useful to consider the biomarkers addressed in the present study for early detection and better treatment of endometriosis. Despite the limited sample size, the present study showed that the combination of enolase-Cr, CA125, and CA19-9 levels enhances the individual diagnostic power of these tests for the detection of endometriosis. However, larger studies will be needed to evaluate the diagnostic potential of this combination.

## Figures and Tables

**(a):** 

Variable	Endometriosis group (*n* = 47)		Controls (*n* = 37)		*p* value
	Min.-max.	Means ± SD	Min.-max.	Means ± SD	
Age (years)	18-44	32.4 ± 6.2	17-40	32.1 ± 7.3	0.942^∗^
BMI (kg/m^2^)	18-34	25.2 ± 3.8	18-40	26.7 ± 4.8	0.194^∗^
Cr	25-557	173^†^ (113) ^††^	35-601	147^†^ (133) ^††^	0.885^∗^

**(b):** 

		Frequency (percent)	Frequency(percent)	
Marital status	Single	7 (14.9)	4 (10.8)	0.415^∗∗^
	Married	40 (85.1)	33 (89.2)	
Gravidity	0	23 (48.9)	11 (29.7)	0.199^∗∗^
	1	11 (23.4)	11 (29.7)	
	2-5	13 (27.7)	15 (40.5)	
Parity	0	24 (51.1)	13 (35.1)	0.201^∗∗^
	1	14 (29.8)	11 (29.7)	
	2-5	9 (19.1)	13 (35.1)	
Abortion	0	41 (87.2)	32 (86.5)	0.585^∗∗^
	1-2	6 (12.8)	5 (13.5)	
Smoking	Yes	0 (0)	1 (2.8)	0.440^∗∗^
	No	47 (100)	36 (97.2)	
Stage of endometriosis	1	1 (2.1)	—	—
	2	7 (14.9)	—	
	3	12 (25.5)	—	
	4	27 (57.5)	—	
Menstrual phase	Follicular phase	29 (61.7)	20 (54)	0.511^∗∗^
	Luteal phase	18 (38.3)	17 (46)	
Menstrual pattern	Regular	29 (61.7)	21 (56.7)	0.806^∗∗^
	Irregular	17 (36.2)	16 (43.3)	
	Amenorrhea	1 (2.1)	0	
Infertility	Yes	19 (40.4)	12 (32.4)	0.501^∗∗^
	No	28 (59.6)	25 (67.6)	

^∗^Results of the Mann-Whitney *U* test; ^∗∗^results of the chi-square test or the Fisher exact test, ^†^ median, ^††^ IQR.

**Table 2:** Urine and blood markers in patients with endometriosis and controls.

Group	*N*		Urinary enolase 1 ng/cc	Enolase-Cr ng/mg of Cr	CA125 (IU/mL)	CA19-9 (IU/mL)
Endometriosis	47	Median (1^st^, 3^rd^ quartiles)	1300.9 (757.5,2434.2)	8.2 (4.8,18.6)	62.0 (33.3,162.0)	10.4 (1.5,45.0)
		Min-max	68.5-8250	026-105	5-655	0.1-138
Controls	37	Median (1^st^, 3^rd^ quartiles)	1132.5 (434.2,1754.4)	6.98 (3.0,11.4)	11.0 (6.59,19.0)	3.0 (1.25,9.93)
		Min.-max.	23.2-7350	0.34-51	4-407	0.3-31.9
^∗^ *p* value			0.080	0.106	<0.001	0.004

^∗^The Mann–Whitney *U* test was used.

**Table 3:** AUC, sensitivity, specificity, PPV, NPV, +LR, and -LR of individual and combined markers for endometriosis.

Marker	ROC-AUC	Sensitivity	Specificity	Positive predictive value	Negative predictive value	Positive likelihood ratio	Negative likelihood ratio
		(95% CI)					
Enolase-Cr	0.443 (0.317-0.569)	97.8 (88.7-99.9)	13.5 (4.5-28.8)	58.9 (55.94-62.27)	83.3 (36.91-99.26)	1.13 (0.8-52.0)	0.16 (0.1-1.0)
CA125	0.220 (0.117-0.322)	69.5 (54.2-82.3)	86.5 (71.2-95.5)	86.4 (73.37-93.6)	69.5 (59.14-78.23)	5.11 (1.8-5.5)	0.35 (0.08-0.4)
CA19-9	0.359 (0.241-0.476)	27.6 (15.6-42.6)	100 (90.5-100.0)	100 (90.1-100.0)	52.1 (47.74-56.52)	—	0.72 (0.2-1.6)
Enolase/Cr + CA125 + CA19 − 9	0.675 (0.573-0.710)	65 (56.5-72.9)	66.6 (57.1-75.3)	71 (64.69-76.57)	60.1 (53.82-66.28)	1.94 (1.5-5.5)	0.52 (0.4-0.7)

The cut-off values for urinary enolase 1, enolase-Cr, CA125, and CA19-9 were 1181 ng/mL, 0.96 ng/mg Cr, 35 IU/mL, and 37 IU/mL, respectively. Abbreviations: ROC-AUC: areas under the receiver operating characteristic curves.

**Table 4:** Individual and combined markers of endometriosis: ROC-AUCs for the severity of endometriosis (*n* = 47).

Variable		*N* (%)	ROC-AUC			
			Enolase-Cr	CA125	CA19-9	Enolase/Cr + CA125 + CA19 − 9
Stage of endometriosis	Stage I + II	8 (17)	0.480 (0.283-0.678)	0.516 (0.345-0.716)	0.470 (0.206-0.683)	0.690 (0.492-0.839)
	
	Stage III + IV	39 (83)	0.601 (0.468-0.756)	0.653 (0.461-0.700)	0.589 (0.500-0.739)	0.763 (0.578-0.878)

Abbreviations: ROC-AUC: area under the receiver operating characteristic curves.

**Table 5:** Marker levels and clinical characteristics of patients with endometriosis (*n* = 47).

Variable		*N* (%)	Urinary enolase 1 ng/mL	Enolase-Cr (ng/mg Cr)	CA125 (IU/mL)	CA19-9 (IU/mL)
			Mean ± SD			
Age (years)	<30	17 (36.2)	1672 ± 467	19 ± 6.5	129.3 ± 33.3	34.2 ± 10
	30–39	20 (42.6)	1668 ± 288	12.8 ± 3.2	129.8 ± 35.4	27.4 ± 8.4
	≥40	10 (21.3)	2585 ± 626	14 ± 2.6	62.7 ± 20.9	18.8 ± 6.5
*p* value		—	0.610^∗^	0.567^∗^	0.378^∗^	0.509^∗^
BMI (kg/m^2^)	<24.9	20 (42.6)	2125 ± 454	15.69 ± 5.5	114 ± 27.9	39.8 ± 9.9
	≥24.9	27 (57.4)	1672 ± 272	15.11 ± 2.6	116 ± 28.1	19.3 ± 5
*p* value		—	0.493^∗∗^	0.345^∗∗^	0.420^∗∗^	0.176^∗∗^
Stage of endometriosis	Stage I + II	8 (17)	1727 ± 943	11.47 ± 6.2	104.8 ± 42.4	15.9 ± 9
	Stage III + IV	39 (83)	1933 ± 254	16.16 ± 3.1	117.5 ± 22.5	30.5 ± 6
*p* value		—	0.819^∗∗^	0.575^∗∗^	0.795^∗∗^	0.274^∗∗^
CA125 (35 U/mL)	≤35	14 (29.8)	1948 ± 553	16 ± 4.4	—	5.46 ± 0.98
	>35	33 (70.2)	1814 ± 269	15 ± 3.5	—	34.27 ± 11.3
*p* value		—	0.567^∗∗^	0.378^∗∗^	—	<0.001^∗∗^
Menstrual phase	Follicular phase	29 (61.7)	1430 ± 207	11.3 ± 2.5	135.7 ± 29.2	28.1 ± 5.9
	Luteal phase	18 (38.3)	2565 ± 523	21.8 ± 5.8	82.6 ± 20.3	28 ± 10.1
*p* value		—	0.017^∗∗^	0.039^∗∗^	0.091^∗∗^	0.712^∗∗^
Infertility	Yes	19 (40.4)	2101 ± 420	16.9 ± 3.8	93.2 ± 19.1	26.2 ± 7.6
	No	28 (59.6)	1704 ± 305	14.3 ± 3.9	130 ± 30.7	29.3 ± 7.3
*p* value		—	0.480^∗∗^	0.503^∗∗^	0.422^∗∗^	0.885^∗∗^

^∗^Results of the ANOVA test; ^∗∗^results of the Mann-Whitney *U* test.

## Data Availability

The datasets used and analyzed during the current study are available from the corresponding author on reasonable request.
